# Orsay Virus CP-δ Adopts a Novel β-Bracelet Structural Fold and Incorporates into Virions as a Head Fiber

**DOI:** 10.1128/JVI.01560-20

**Published:** 2020-10-14

**Authors:** Yusong R. Guo, Yanlin Fan, Ying Zhou, Miao Jin, Jim L. Zhang, Hongbing Jiang, Matthew V. Holt, Tao Wang, Nicolas L. Young, David Wang, Weiwei Zhong, Yizhi J. Tao

**Affiliations:** a Department of BioSciences, Rice University, Houston, Texas, USA; b Verna & Marrs McLean Department of Biochemistry & Molecular Biology, Baylor College of Medicine, Houston, Texas, USA; c Department of Molecular and Cellular Biology, Baylor College of Medicine, Houston, Texas, USA; d Department of Molecular Microbiology and Pathology & Immunology, Washington University, School of Medicine, St. Louis, Missouri, USA; University of Kentucky College of Medicine

**Keywords:** Orsay virus, crystallography, *Caenorhabditis elegans*, β-bracelet, viral fiber

## Abstract

Fiber proteins are commonly found in eukaryotic and prokaryotic viruses, where they play important roles in mediating viral attachment and host cell entry. They typically form trimeric structures and are incorporated into virions via noncovalent interactions. Orsay virus, a small RNA virus which specifically infects the laboratory model nematode Caenorhabditis elegans, encodes a fibrous protein δ that can be expressed as a free protein and as a capsid protein-δ (CP-δ) fusion protein. Free δ has previously been demonstrated to facilitate viral exit following intracellular expression; however, the biological significance and prevalence of CP-δ remained relatively unknown. Here, we demonstrate that Orsay CP-δ is covalently incorporated into infectious particles, the first example of any attached viral fibers known to date. The crystal structure of δ(1–101) (a deletion mutant containing the first 101 amino acid [aa] residues of δ) reveals a pentameric, 145-Å long fiber with an N-terminal coiled coil followed by multiple β-bracelet repeats. Electron micrographs of infectious virions depict particle-associated CP-δ fibers with dimensions similar to free δ. The δ proteins from two other nematode viruses, Le Blanc and Santeuil, which both specifically infect Caenorhabditis briggsae, were also found to form fibrous molecules. Recombinant Le Blanc δ was able to block Orsay virus infection in worm culture and vice versa, suggesting these two viruses likely compete for the same cell receptor(s). Thus, we propose that while CP-δ likely mediates host cell attachment for all three nematode viruses, additional downstream factor(s) ultimately determine the host specificity and range of each virus.

**IMPORTANCE** Viruses often have extended fibers to mediate host cell recognition and entry, serving as promising targets for antiviral drug development. Unlike other known viral fibers, the δ proteins from the three recently discovered nematode viruses are incorporated into infectious particles as protruding fibers covalently linked to the capsid. Crystal structures of δ revealed novel pentameric folding repeats, which we term β-bracelets, in the intermediate shaft region. Based on sequence analysis, the β-bracelet motif of δ is conserved in all three nematode viruses and could account for ∼60% of the total length of the fiber. Our study indicated that δ plays important roles in cell attachment for this group of nematode viruses. In addition, the tightly knitted β-bracelet fold, which presumably allows δ to survive harsh environments in the worm gut, could be applicable to bioengineering applications given its potentially high stability.

## Abstract (precis)

Viruses often have extended fibers to mediate host cell recognition and entry, serving as promising targets for antiviral drug development. Unlike other known viral fibers, the δ proteins from the three recently discovered nematode viruses are incorporated into infectious particles as protruding fibers covalently linked to the capsid. Crystal structures of δ revealed novel pentameric folding repeats, which we term β-bracelets, in the intermediate shaft region. Based on sequence analysis, the β-bracelet motif of δ is conserved in all three nematode viruses and could account for ∼60% of the total length of the fiber. Our study indicated that δ plays important roles in cell attachment for this group of nematode viruses. In addition, the tightly knitted β-bracelet fold, which presumably allows δ to survive harsh environments in the worm gut, could be applicable to bioengineering applications given its potentially high stability.

## INTRODUCTION

Discovered in 2011, Orsay virus is a small, positive-sense RNA (+RNA) virus that infects the terrestrial nematode Caenorhabditis elegans (C. elegans). Infection specifically targets the intestine, causing morphological changes such as intermediate filament disorganization and nuclear degeneration (1). Since the discovery of Orsay, two other nematode-infecting viruses termed Santeuil and Le Blanc have also been identified, infecting the closely related *Caenorhabditis briggsae* (C. briggsae). The genome of these three nematode viruses consists of two RNA segments, termed RNA1 and RNA2, containing a total of three open reading frames (ORFs). RNA1 encodes an RNA-dependent RNA polymerase (RdRP), while RNA2 encodes a capsid protein (CP) and a non-structural protein δ. Phylogenetic analyses indicated that all three nematode viruses are remotely related to nodaviruses, a family of bi-segmented, +RNA viruses known to infect insects and fish (2). The evolutionary relationship was later confirmed when the structure of the Orsay capsid was solved (3), exhibiting T = 3 icosahedral symmetry with trimeric spikes similar to those found in beta-nodaviruses (4).

Despite a shared evolutionary lineage with nodaviruses, the three nematode viruses exhibit several distinct features, including the use of an ATG-independent mechanism for translation initiation and the lack of B1/B2 proteins involved in suppression of the innate immune response (5–7). Additionally, the δ ORF, which is conserved in all three nematode viruses, is absent from nodaviruses. A CP-δ fusion protein has also been detected during Orsay infection, presumably due to translational read-through at an RNA stem-loop functioning as an intergenic ribosomal frameshift signal (7). This RNA stem-loop was also predicted in the Santeuil and Le Blanc viruses (7), suggesting that CP-δ is also expressed during Santeuil and Le Blanc infection.

Our previous work demonstrated that the Orsay δ protein forms a pentameric fibrous structure (8), with secondary structure mapping indicating a predominantly β-stranded intermediate shaft domain. In transgenic worms, δ was found to specifically target the apical surface of the intestine, where it colocalizes with the terminal web (9). When single point mutations were introduced to disrupt the translation of CP-δ, the resulting mutant virus was found to be significantly less infectious. In addition, lone CP-δ was noted to form inclusion bodies; but when coexpressed with CP, recombinant capsids were observed with protruding fibers, suggesting that CP-δ may be an intrinsic component of native viral capsids (8). Furthermore, addition of recombinant, free δ to viral filtrate functioned as a competitive inhibitor to infection, supporting the overall idea that CP-δ functions to mediate host entry (8).

Virion-associated fibers are commonly found in non-enveloped viruses infecting organisms ranging from bacteria to humans. These fibers assume a variety of functions, such as stabilizing the viral capsid (10, 11), recognizing host receptors (12), facilitating virus attachment (13), and mediating host cell entry (14, 15). For viruses infecting vertebrate hosts, viral fibers are often primary targets for neutralizing antibodies, as they are well exposed on capsid surfaces (16, 17). These fibers often assume trimeric structures (18) that are either α-helical or β-stranded. The α-helical fibers can have the structure of a trimeric coiled coil or a helix-turn-helix supercoil, as seen in bacteriophage T4 fibritin (19) and bacteriophage ϕ29 head fiber (13), respectively. A variety of β-stranded fibers have also been observed, including the β-spirals in adenovirus and reovirus (20–23), the β-helices in bacteriophage T4 tail needle (24, 25), and the six-stranded antiparallel sheet in the T4 long tail fiber (26). Interestingly, the Mimivirus fiber, which promotes host phagocytosis with bacterial polysaccharide mimics, adopts a unique collagen type structure (27). Studies of viral fibers have not only provided functional insights into viral infection cycles, but also enriched our understanding of protein folds and their stabilities. Given this context, some of the major questions regarding the Orsay δ include: (i) what kind of β structure does it have; (ii) is CP-δ indeed part of the infectious particle; and (iii) if so, what role does the CP-δ fiber play in determining host specificity for the three nematode viruses?

To address these questions, we purified infectious Orsay particles and confirmed the existence of capsid-associated fibers through electron microscopy (EM). Western blot analysis indicated an estimated stoichiometry of approximately one pentameric fiber per capsid. We then determined the crystal structure of an Orsay δ truncation mutant, namely, δ(1–101), containing the first 101 amino acid residues. Upon analyzing the structure, it was revealed that immediately following the N-terminal α-helical coiled coil, the δ fiber adopts multiple repeats of a novel β-bracelet fold predicted to constitute the majority of the fiber shaft. The δ proteins from the other two nematode-infecting viruses Le Blanc and Santeuil were also expressed and found to form fibrous molecules under EM, suggesting similar structures and overall conserved functions. To examine the role of δ in determining host specificity, the recombinant δ protein of each of the three nematode viruses was supplemented in *trans* to test its ability to inhibit the infection of the orthologous virus versus the other two viruses. It was found that recombinant Le Blanc δ was able to block Orsay virus infectivity and vice versa, indicating that these two viruses can bind to the same receptor molecule. The fact that these viruses ultimately infect different nematode species suggests that their host range is not solely restricted by δ-mediated host cell attachment, and is instead determined by multiple downstream factors.

## RESULTS

### Infectious Orsay particles contained protruding fibers.

**FIG 1:**
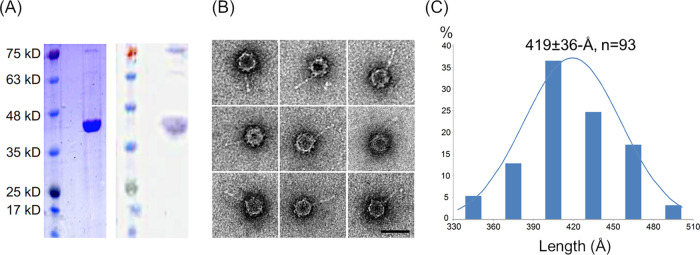
Infectious Orsay particles associated with δ-like fibers. (A) SDS-PAGE and Western blot of purified infectious Orsay particles. (B) Negative staining TEM images of infectious Orsay particles. All nine particles contain a long fiber with a slightly enlarged terminal domain. Scale bar, 50 nm. (C) Length measurements for virion-associated δ fibers.

We previously observed that the Orsay CP-δ fusion protein incorporates into recombinant virus-like particles (VLPs) when coexpressed with CP in insect cells (8). To confirm that the CP-δ fusion protein is indeed an integral part of the authentic virion, we purified infectious Orsay particles from the culture supernatant of Orsay-infected *rde-1* worms grown in liquid suspension (7). The RNAi defective *rde-1* strain was chosen for viral propagation as they are able to support higher levels of infection compared to wild-type N2 worms. After applying the concentrated culture supernatant to an iodixanol gradient, Orsay particles were banded to an ∼1.3 g/ml position. The resulting virion samples were then analyzed by Western blotting with anti-CP antiserum. Two protein species were clearly visible, with a heavy band at ∼36 kDa corresponding to the viral CP and a much weaker band at ∼72 kDa corresponding to the CP-δ fusion protein (Fig. 1A). Assuming a T = 3 icosahedral symmetry and considering the calculated molecular weights of CP and CP-δ, we estimated that there should be ∼1.1 ± 0.7 pentameric CP-δ fibers in each viral particle based on the Western blot band intensities (Fig. 1A).

When purified virions were subjected to negative-staining transmission EM (TEM), spherical particles were observed with a diameter of ∼360 Å (Fig. 1B), consistent with dimensions reported by Félix et al. (1) and Jiang et al. (7) for infectious Orsay particles and by Guo et al. (3) for recombinant Orsay capsids. These particles have a spiky appearance, presumably due to the presence of the 60 trimeric protrusions on the quasi 3-fold symmetry axes. Most interestingly, long fibers that closely resemble filamentous δ molecules were clearly seen on viral particles (Fig. 1B). To confirm that these fibrous structures were not merely genomic RNA released from Orsay particles, purified virion samples were treated with RNase A, but the appearance of the long fibers did not change. DNase I treatment also had no effect on the particle appearance, indicating that these virion-associated fibers were likely made of protein.

The length of these virion-associated fibers was measured to be 419 ± 36 Å (Fig. 1C), closely matching the previously measured length of free δ fibers at 419 ± 52 Å (8), as well as fibers protruding from the recombinant Orsay VLPs measured at 387 ± 42 Å (8). Analysis of EM micrographs indicated approximately 19% of the observed Orsay particles (i.e., 93 out of 479 particles) had a single fiber. None were observed to have two or more fibers. In spite of the calculated stoichiometry of ∼1.1 fibers per capsid, the observation of pentameric fibers in only ∼20% of the capsids is likely due to the orientation of the particles and/or negative staining artifacts.

### Crystal structure of an N-terminal Orsay δ(1–101) fragment.

**FIG 2:**
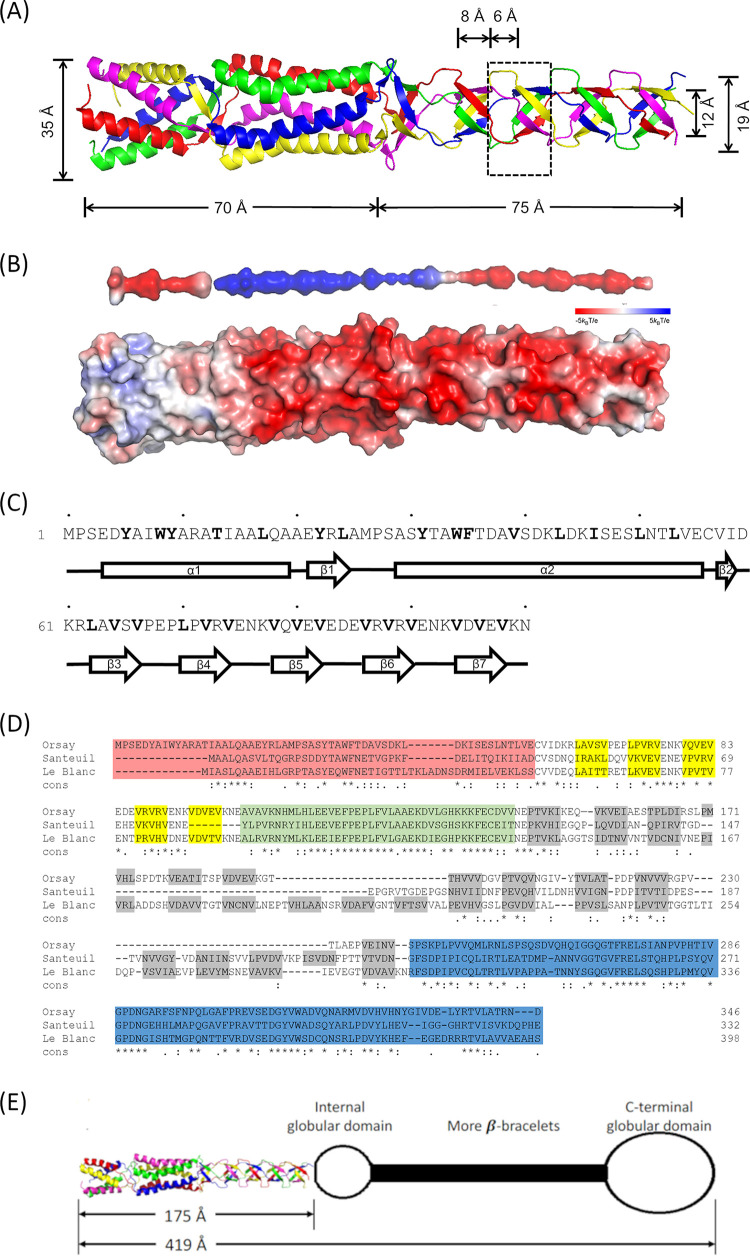
Crystal structure (1.8 Å) of Orsay δ(1–101) and structure prediction of δ. (A) Ribbon diagram. The five subunits are shown in different colors. (B) Surface representation. The interior surface (top) and exterior surface (bottom) are both colored by electrostatic potential. (C) Secondary structure assignment for one polypeptide chain. Bold letters highlight hydrophobic residues facing the fiber interior. α-helices are shown by boxes, β-strands by arrows, nonstructured loops by lines. (D) Multisequence alignment of δ proteins from all three nematode viruses. Alignment was generated by T-Coffee (45). The N-terminal α-helical bundle domain is highlighted in red and β-bracelet segments in yellow. The predicted internal globular domain is highlighted in light green; the predicted β-bracelet segments are in gray and the predicted C-terminal globular domain is in light blue. (E) Structural model of Orsay δ.

Pentameric protein fibers are rarely observed in viruses. To better understand the β structure fold of δ, we crystallized δ(1–101), a δ deletion mutant containing the first 101 amino acid residues. Subsequent molecular replacement with δ(1–66) as a model allowed structural determination of δ(1–101) at a resolution of 1.8 Å (Fig. 2). Each crystallographic asymmetric unit of the P2_1_2_1_2_1_ space group was found to contain five polypeptide chains (i.e., A, B, C, D, and E) that fold into a fibrous-shaped molecule. Electron density was well defined for the entire sequence, except for residue 101 in chain E.

**FIG 3:**
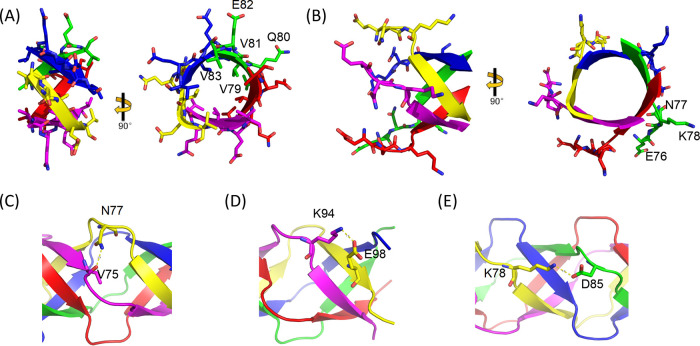
Orsay δ(1–101) exhibits a novel pentameric β-bracelet fold. (A) Ribbon diagram of one β-bracelet segment (residues 79 to 83), with side chains shown in sticks. (B) Ribbon diagram of one connecting loop (residues 76 to 78). Side chains are shown in sticks. Both a side view (left) and a top view (right) are provided in (A) and (B). The residues in the chain colored green are labeled. (C to E) Three different forms of side chain interactions involving loop residues at different positions.

The resulting crystal structure of δ(1–101) revealed a novel β structure that is distinct from both β spirals and β helices. The α-helical bundle previously observed in δ(1–66) is maintained in δ(1–101), with the first α-helix consisting of residues 4 to 20 and the second α-helix consisting of residues 30 to 56 (Fig. 2A and B). The second α-helix has a large kink at residue 36 due to a single-residue insertion in the heptad repeat (Fig. 2C). Starting from residue 63, the polypeptide chain assumes a series of five-residue long β-strands that intertwine with the other polypeptides of the pentamer to form small, right-handed β-barrels which we term “β-bracelets” (Fig. 2A, Fig. 3A). Adjacent β-bracelets are connected by short loops that are three-residues long. δ(1–101) contains five such β-bracelet repeats, which cover a length of ∼75 Å. Each bracelet repeat has a diameter of ∼21 Å (12 Å measured from main chain) and a height of ∼8 Å (Fig. 2A). The loops connecting two neighboring bracelet repeats bulge out with a diameter of ∼21 Å (19 Å measured from main chain) and a height of ∼6 Å (Fig. 2A). The fiber shaft corresponding to the β-bracelet region is much thinner compared to the N-terminal coiled coil regions (21 Å versus 35 Å), as the central channel running throughout the δ(1–101) molecule varies in diameter from 3 to 5 Å (Fig. 2B).

As a novel structural fold, the β-bracelet from δ(1–101) is associated with highly characteristic amino acid sequences. The short β-strands typically bear a consensus sequence of (V/L)x_2_Vx_4_V, where V is valine, L is leucine, and x is often a small, polar or charged amino acid (Fig. 2C). The hydrophobic side chains of leucine and valine face the interior of the bracelet motif, maintaining the hydrophobicity of the core throughout the fiber (Fig. 3A). Each β-strand participates in five alternating main-chain hydrogen bonds with two neighboring molecules. The connecting loops invariantly contain three residues with a consensus sequence of y_1_(E/D/N)y_3_, where y is either a positively or negatively charged residue (Fig. 2C). Sidechains from the residues y_1_ and y_3_ often point into solvent, whereas sidechains from the middle E/D/N residues often point toward the adjacent chain, forming hydrogen bonds with a main-chain carbonyl (Fig. 3B and C). It is also common for a loop y_3_ sidechain to point toward the C-terminal end of the polypeptide, forming salt bridges with either an x_4_ sidechain from the β-bracelet in the adjacent chain (Fig. 3D) or with a loop y_2_ residue sidechain in the next chain over (Fig. 3E). Unlike the N-terminal helical bundle, no water-like density was observed in the central channel of the β-bracelet region, which is entirely hydrophobic.

### Comparison of δ(1–101) to other β structures.

**FIG 4:**
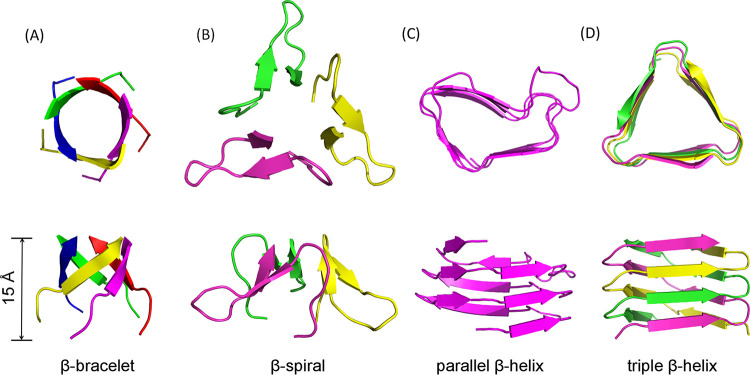
Comparison of β fiber structure motifs. (A) β-bracelet. Orsay δ protein residues 76 to 83 are shown. (B) β-spiral. Adenovirus fiber protein (PDB ID: 1QIU, residues 359 to 375). (C) Parallel β-helix. P22 tailspike protein (PDB ID: 1TSP, residues 429 to 502). (D) Triple β-helix. T4 phage gp5 (PDB ID: 4JJ2, residues 484 to 514). Top panels and bottom panels in (A to D) are for top views and side views, respectively.

β filaments are frequently observed in viruses, but their structural folds can differ substantially. Both adenovirus and reovirus are well-known examples of viruses with capsid-associated fibers. However, unlike Orsay δ, the β-stranded shafts in both adenovirus and reovirus are made of trimeric β-spiral repeats (Fig. 4A and B) (20, 28). Each β-spiral consists of a pair of anti-parallel β-strands linked with a tight turn that is usually mediated by proline or glycine (29). As a result, the β-spiral is stabilized by both interchain and intrachain hydrogen bonds. In addition, each β-spiral contains not only a hydrophobic core, but also hydrophobic patches on the outer surface (22).

The Orsay β-bracelet is also structurally distinct from β-helices, another β-structural motif consisting of helical β strands. Two major types of β-helices have been reported, parallel β-helices, as seen in the tail spike protein of phage P22 (25) (Fig. 4C), and triple β-helices, as observed in gp5C of the T4 tail lysozyme complex (30) (Fig. 4D). Parallel β-helices utilize only one polypeptide chain to form an entire helix of various turns with one to four faces, i.e., antifreeze protein (31), alkaline protease (32), P22 tailspike (25), and fluoroquinolone resistance protein (33). In addition, each face of a parallel β-helix consists of parallel β-strands stacked on top of each other, which can further assemble into oligomers via extensive intersubunit contacts (25). On the other hand, a triple β-helix consists of a right-handed coil of parallel β-strands formed by three polypeptide chains (Fig. 4D) in a triangular arrangement. Each of these polypeptide chains participates in hydrogen bonding with its neighbors in addition to positioning aliphatic side chains toward the interior of the helix, thus forming a hydrophobic core to overall stabilize the helical assembly.

β-bracelets from the Orsay δ protein exhibit several unique characteristics compared to β-spirals and β-helices (Fig. 4). In terms of multimeric state, β-bracelets are pentameric while β-spirals and β-helices are either trimeric or monomeric. Additionally, β-strands within a β-bracelet run at an ∼45° angle relative to the central axis; this angle in β-helices is approximately 90°, whereas in β-spirals the angles are ∼20° and ∼120° for the two antiparallel β-strands, respectively. Finally, the β-bracelet has an alternating arrangement of β-strands and loops of fixed lengths and distinct sequence motifs, forming a roughly circular assembly.

A DALI search was subsequently performed to search for δ(1–101) structural homologs against the whole Protein Data Bank database. However, all meaningful hits were aligned to the coiled coil region of δ(1–101) instead of the β-bracelets. Interestingly, it was noted that porins contain similarly circular-shaped β-barrel structures that appear to be somewhat similar to the β-bracelet fold. However, these structures are monomeric and much shorter in height. Porins are also much larger, containing 8 to 22 β strands compared to the 5 found in β-bracelets. Considering these differences, it is unlikely that porins and β-bracelets share common evolutionary origins or have similar functions.

### Additional β-bracelet repeats were predicted in Orsay δ.

The conserved sequence pattern of the β-bracelet motifs from δ(1–101) allowed us to make structure predictions for the rest of the δ protein. We found that the consensus sequence of (L/V)x_2_Vx_4_V stops after residue 102, but then resumes from residue 146 to 249 in a more variable form of φ_1_x_2_φ_3_x_4_φ_5_, where φ is usually hydrophobic (Fig. 2D). In total, there may be up to 11 additional β-bracelet segments that are separated by loops of various lengths. These 11 β-bracelet segments should give rise to an estimated length of ∼165 Å, assuming each segment has a height of ∼15 Å (Fig. 2A). In line with this, the internal globular domain is predicted to consist of residues 103 to 145, while the C-terminal globular domain likely consists of residues 250 to 346. Based on this organization, the length of the composite Orsay δ fiber should be the sum of 145 Å (the length of δ[1–101]), 165 Å (11 β-bracelet segments), 50 Å (the diameter of the C-terminal globular head), and 30 Å (the diameter of the internal globular domain), totaling 390 Å and mirroring the estimated length of 419 Å based on negative staining EM images (Fig. 1, Fig. 2E).

### Characterization of δ fibers from Le Blanc and Santeuil viruses.

**FIG 5:**
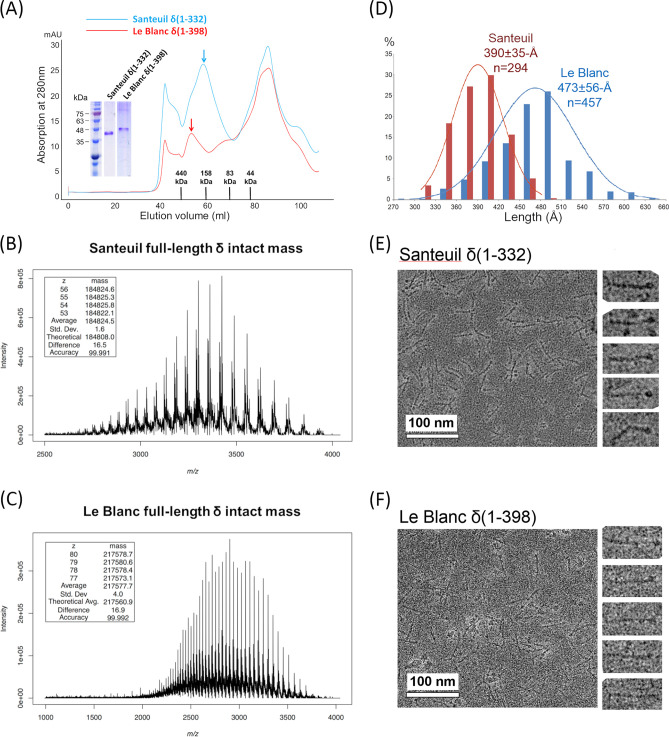
Analysis of Santeuil and Le Blanc δ proteins. (A) Purification of recombinant δ proteins from Le Blanc and Santeuil viruses. Gel filtration chromatogram using a Superdex-200 column showed an eluted peak corresponding to an apparent molecular weight of more than 158 kDa for both Le Blanc and Santeuil δ with SDS-PAGE analysis depicting the two purified δ proteins. (B and C) Mass spectra of Santeuil δ (B) and Le Blanc δ (C). Both spectra only depicted masses consistent with a pentameric assembly for δ. No peaks suggestive of monomers or other oligomeric states were observed. (D) Length measurements for the two full-length δ fibers. (E and F) TEM images of Santeuil and Le Blanc δ proteins by negative staining. On the right are five enlarged δ aligned in the horizontal direction. Scale bar, 100 nm.

Next, we sought to determine whether the structure and function of δ are conserved in all three known nematode viruses, Orsay, Santeuil, and Le Blanc. Pairwise sequence alignment between the respective δ proteins of each virus indicates ∼39% identity between Orsay and Le Blanc, ∼37% between Orsay and Santeuil, and ∼39% between Le Blanc and Santeuil (Fig. 2D). To characterize the structure of δ from Le Blanc and Santeuil viruses, their coding sequences were each cloned into a pET vector for overexpression in E. coli. In both cases, recombinant δ was expressed as a soluble protein and more than 50% could be recovered in the cytoplasmic fraction. When subjected to a Superdex-200 gel filtration column, a sharp peak at ∼53 ml and ∼58 ml was observed for Le Blanc and Santeuil δ, respectively (Fig. 5A). An elution volume in this range corresponds to an apparent molecular mass of more than 158 kDa.

Subsequent analysis via mass spectrometry confirmed that full-length Le Blanc δ forms a pentamer with an observed mass of 217,577.7 Da, nearly identical to the predicted theoretical mass of 217,560.9 Da (monomer theoretical mass: 43,512.2 Da) (Fig. 5B). Additional minor peaks were observed that deviated by −526.9 Da (0.24% difference) and 294.0 Da (0.14% difference), which also suggested a pentameric complex. No major peaks were observed that would account for any other multimeric configuration. Likewise, mass spectrometry analysis confirmed that full-length Santeuil δ also forms a pentamer (Fig. 5C). The observed mass of the Santeuil δ complex is 184,824.5 Da, while the theoretical pentamer mass is 184,808.0 Da (monomer theoretical mass: 36,961.6 Da).

Analysis with negative-staining TEM indicated both Le Blanc and Santeuil δ formed fibrous molecules (Fig. 5E and F). Based on measurements performed on more than two hundred particles, the length of Santeuil and Le Blanc δ was determined to be ∼390 ± 35 Å and ∼473 ± 56 Å, respectively (Fig. 5D). This variation in δ fiber length between the three nematode viruses is likely due to differences in the number of β-bracelet segments. Nonetheless, a conserved globular head domain with a diameter of ∼50 Å was consistently observed at the ends of all three fibers, likely formed by the C-terminal region of the polypeptide as previously noted in Orsay δ (8) (Fig. 2D). However, the smaller internal globular domain observed in Orsay δ was missing in both Le Blanc and Santeuil fibers, despite sequences corresponding to this domain being present in all three viruses (Fig. 2D). Furthermore, a bending point was frequently observed near the middle of the Santeuil fibers (Fig. 5E) but not in the fibers of the other two nematode viruses.

### The effect of orthologous δ proteins on Orsay host entry.

**FIG 6:**
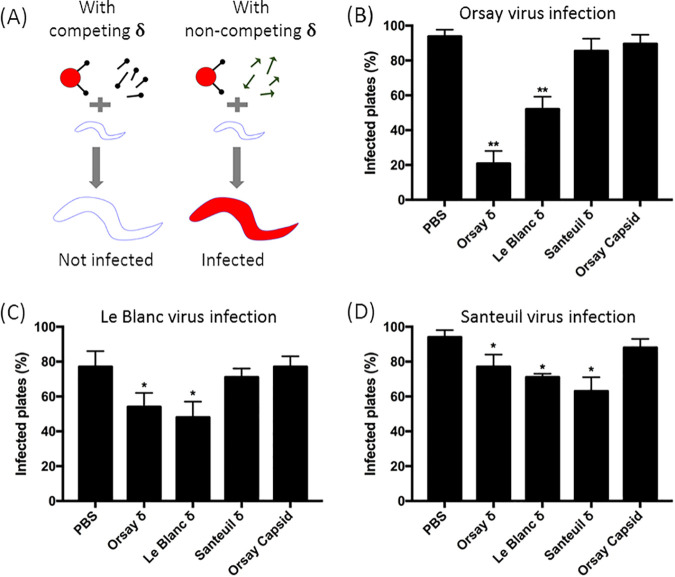
δ protein competition assay. (A) Schematic drawing of the experimental design. Worms, either C. elegans or C. briggsae, were exposed to infectious virions and the δ proteins of each respective nematode virus. If a δ protein does indeed bind to the same host cell receptor as Orsay, then subsequent infectivity should be reduced via competitive inhibition, as shown in the left panel. Conversely, if a δ does not bind competitively to host cell receptors, infectivity should remain unaffected, as shown in the right panel. (B to D) Results for the δ protein competition assay with Orsay, Le Blanc, and Santeuil viruses, respectively. (B) C. elegans-Orsay infection was found to be significantly inhibited with addition of free Orsay and Le Blanc δ. (C) C. briggsae-Le Blanc infection exhibited similar, but milder patterns of inhibition with the addition of free Orsay and Le Blanc δ, suggesting the two viruses share a host receptor. (D) C. briggsae-Santeuil infection was only inhibited with addition of free Santeuli δ, suggesting use of an entirely separate host cell receptor.

Because CP-δ has been found to function in mediating viral attachment to host cells (8) and likely binds to host receptors through its globular C-terminal domain, we sought to determine whether CP-δ dictates the overall host range of each respective nematode virus. To test this, we added δ proteins from different nematode viruses to the culture medium and examined their impact on Orsay infection in a protein-competition assay. If an orthologous δ protein does indeed compete against Orsay CP-δ for receptor binding sites, then the efficiency of Orsay infection should be significantly reduced (Fig. 6A). To conduct the assay, we first determined the viral titer of prepared Orsay samples and chose the lowest viral concentration capable of maintaining over 70% infectivity. At this viral concentration, adding full-length Orsay δ to the culture medium at a final concentration of 0.18 nM reduced Orsay infectivity from 93% to 20% (Fig. 6B). Interestingly, adding full-length Le Blanc δ at the same concentration also significantly reduced Orsay infectivity from 93% to 52% (*P < *0.05, Student’s *t* test). Adding Santeuil δ, however, had no significant effect on Orsay infectivity, comparable to the negative control (Fig. 6B). These results suggest that Le Blanc δ is able to block Orsay host entry, possibly by competitively binding to the same host cell receptor(s) utilized by Orsay δ.

To determine whether Orsay δ protein can reciprocally inhibit Le Blanc infection, we mixed purified, free Orsay δ with Le Blanc viral filtrate and infected groups of C. briggsae. Le Blanc-infected C. briggsae exhibit a more transparent body, thus allowing quantification of viral infection. As expected, free Orsay δ was nearly as effective as free Le Blanc δ in reducing Le Blanc virus infectivity, while the Santeuil δ and the Orsay capsid were found to have negligible effects similar to the negative control (Fig. 6C). When the three δ proteins were applied to a similar experiment assessing for impacts on Santeuil virus infection, free Santeuil δ had significant effects in reducing infectivity (Fig. 6D), as expected, while both Orsay δ and Le Blanc δ exhibited much milder inhibitory effects in comparison (Fig. 6D).

In summary, the protein competition assay suggests that Le Blanc δ is most similar to Orsay δ in terms of their receptor binding activity. However, the fact that the Le Blanc virus is ultimately unable to infect C. elegans indicates that there are other downstream steps following viral attachment that ultimately determine the host range of each respective virus. Furthermore, although Le Blanc and Santeuil infect the same host, the fact that each respective δ protein had no inhibitory effect on the infection rates of the other virus suggests they likely utilize different host receptors for attachment and entry.

## DISCUSSION

The observation of δ fibers in infectious Orsay particles not only confirms its biological relevance, but also demonstrates that the structural architecture of δ is preserved in both the free protein and the fusion protein. Stoichiometric calculation based on Western blot band densities of purified virion samples indicated that there is approximately one fiber in each particle. It is unclear how the CP-δ fiber copy number varies among individual particles, but this stoichiometry was rather constant for at least three different batches of purified viral samples. Only one virus band was observed in the iodixanol density gradient, thus we have not been able to isolate fiberless particles. While this may be due to lack of resolving power, there is probably only a very small percentage of fiberless particles, if such a population does exist at all. Considering the role of CP-δ in host entry, fiberless particles are likely uninfectious. The stoichiometry of one CP-δ fiber per virion is much less than that for recombinant Orsay capsids produced from protein overexpression, where up to eight fibers could be clearly seen in one capsid. The low expression level of CP-δ during natural infection likely explains the low fiber copy number for authentic virions.

Because the δ pentamer is extremely stable (see discussion below), the CP-δ pentamer may function as a nucleation site for capsid assembly, much like the connectors of tailed bacteriophages, where it has been shown that phage ϕ29 connector protein lowers the critical concentration of capsid protein required for assembly (34). Because the Orsay CP dimer is also a stable capsomere in solution, we speculate that each subunit in the CP-δ pentamer further dimerizes with CP molecules to form a pentamer of CP/CP-δ dimers. The biological function of the CP-δ fiber is unlikely to be related to genome release in the same manner as, for instance, the phage connector, as its central channel is less than 5 Å in diameter and thus too small to accommodate either single- or double-stranded nucleic acids. However, it remains possible that the special vertex occupied by the CP-δ fiber has altered stability compared to other vertices and may nonetheless play a special role in genome delivery.

Mass spectrometry indicates that δ fibers from all three nematode viruses form stable pentamers with no evidence suggesting other multimeric states. The ability to survive reverse phase chromatography on a C-3 column without dissociating into monomers is indicative of a strong and robust protein assembly. We also found that the addition of DTT did not reduce pentamers to monomers, indicating that noncovalent interactions play a crucial role in keeping the pentameric assemblies intact. Such high stability may be necessary for the δ fiber to maintain structural integrity in various environments, including the worm gut, which is known to be highly acidic and rich in digestive enzymes (35). The flexibility of the CP-δ fiber could be provided by the short insertion loops connecting the β-bracelets, as these loops are associated with elevated temperature factors based on the δ(1–101) crystal structure.

Because δ is a multidomain protein, the contribution of its different domains (i.e., coiled coil, β-bracelet segments, the internal and the terminal globular domains) to the overall stability and folding of the pentameric fiber cannot yet be accurately assessed. According to our prediction, the β-bracelet segments account for 255 Å of the ∼420 Å long fiber. β-bracelet segments in δ fibers were found to be stabilized by extensive interchain hydrogen bonds, salt bridges, and, most importantly, an extended hydrophobic core. As a novel structural motif, the thermodynamic properties of the β-bracelet motif warrant further investigation. However, it is worth noting that viral trimeric β fiber structures are often highly resistant to heat, denaturants, proteases, and detergents (18), mirroring the inherent properties of the Orsay δ protein. Compared to other β-stranded structures, the β-bracelet motif gives rise to the longest fiber per amino acid residue. For example, to form a 15-Å structure, only 8 residues are required in a pentameric β-bracelet assembly, while trimeric β-spirals and triple β-helices would require 17 and 31 residues, respectively (Fig. 4).

Work remains to determine whether the β-bracelet shaft of Orsay δ assumes any active role in infection, other than acting as a structural support. With their elongated, solvent-accessible surfaces suitable for the binding of flexible ligands, the majority of β-helical and β-spiral structures are found in viral adhesins or bacterial virulence factors—which are involved in the recognition of polysaccharides, lipopolysaccharides, and protein receptors (36, 37). For example, the P22 tailspike protein uses its parallel β-helix domain to selectively recognize polysaccharides (18), while the reovirus σ1 protein has been found to bind sialic acid through its β-spiral repeat (20). The parallel β-helix motif is also found in the actin binding domain of cyclase-associated protein (38), in which the dimerization of β-helices creates a concave surface which potentially binds G-actin. Further investigation on the host cell receptors and the host cytoskeleton reorganization properties of Orsay δ (9) will better delineate how the protein and its repeating β-bracelet motif functions to support Orsay infection.

Overall, several lines of evidence indicate that CP-δ fibers broadly function in Orsay replication by mediating host cell attachment (8). While it is reasonable to assume that the δ protein of each nematode virus determines their host specificity, our findings suggest an alternate scenario. Le Blanc δ is able to reduce Orsay infection, and vice versa, most likely by competing with Orsay virus for the same host receptor. Meanwhile, Santeuil δ had little effect on the infectivity of Orsay and Le Blanc viruses, suggesting that the Santeuil virus utilizes a different receptor than Orsay and Le Blanc. Despite the fact that Orsay and Le Blanc can recognize the same receptor molecule, they remain restricted to their respective nematode host, indicating that host specificity is not determined entirely by δ-mediated host cell attachment. As a result, there is likely another downstream step that ultimately limits the effective replication of Le Blanc virus in C. elegans.

Nematodes are the most abundant animals on this planet, with many being pathogenic and affecting human health as well as agriculture. Results from this study, especially the discovery of the β-bracelet fold and the role of the CP-δ fiber, lay the foundation to further elucidate the life cycle of these nematode viruses and to possibly enable the engineering of δ proteins to target pathogenic nematodes. Additionally, the ability to study virus infection in an intact gut within the transparent body of a worm makes the Orsay-C. elegans system an outstanding *in vivo* model to delineate mechanisms related to the replication and pathogenesis of eukaryotic viruses that may be generally applied to human gastrointestinal diseases caused by rotavirus, norovirus, and astrovirus infection.

## MATERIALS AND METHODS

### Cloning and protein expression.

Genes encoding full-length δ from Orsay (GenBank accession no. HM030971.2), Santeuil (GenBank accession no. HM030973.1) and Le Blanc (GenBank accession no. JQ943580.2) were each inserted into a modified pETDuet-1 vector between the NdeI and XhoI sites with an N-terminal 6×His-SUMO tag. Orsay δ(1–101) was constructed by mutating the residue 102 to a stop codon using PCR. Plasmids with confirmed sequences were transformed into E. coli Rosetta 2 competent cells (Novagen) for protein overexpression. Recombinant protein expression was induced by adding 1 mM isopropyl β-D-1-thiogalactopyranoside (IPTG) when the optical density at 600 nm (OD_600_) reached 0.6 to 0.8. After overnight incubation at 15°C, cells were harvested by centrifugation at 3,700 × *g* for 20 min (Beckman Coulter SX4750) and resuspended in lysis buffer containing 50 mM Tris pH 8.0, 300 mM NaCl, 10% glycerol (vol/vol), 5 mM 2-mercaptoethanol (2-ME), 1 mM NaN_3_, and 1 mM phenylmethylsulfonyl fluoride (PMSF). Cells were then lysed by sonication and debris was removed by centrifugation at 12,000 × *g* for 30 min (Beckman Coulter JA-25.50). The clarified lysate was then loaded on preequilibrated Ni-NTA resin (Thermo Fisher Scientific). The 6×His-SUMO affinity tag on the recombinant proteins were cleaved by mixing eluted protein samples with purified SUMO (Ulp) protease at a 10:1 mass ratio at 4°C. After overnight incubation, samples were brought to 25 mM imidazole by dialysis, and a second Ni-NTA purification step was applied to remove the cleaved 6×His-SUMO tag and the His-tagged SUMO protease, leaving untagged target proteins in the flowthrough. δ was further purified to near homogeneity by a final gel filtration step in 50 mM Tris pH 8.0, 200 mM NaCl, 5 mM 2-ME, and 1 mM NaN_3_ using a Superdex-200 column (GE Healthcare). The peak fractions were combined and concentrated for crystallization, electron microscopy, or mass spectrometry.

### Crystallization and structure determination of of δ(1–101).

Crystals of Orsay δ(1–101) were obtained by hanging drop vapor diffusion by mixing 1.5 μl of protein solution at 5 mg/ml with 0.5 μl of mother liquor containing 0.1 M ammonium sulfate, 0.1 M sodium acetate pH 4.6, and 25% (wt/wt) PEG 4000. Rod-shaped crystals appeared in 3 days after incubation at 20°C and grew to a full length of ∼300 μm in 2 weeks. Crystals were transferred to mother liquor containing 25% glycerol as cryoprotectant and flash-frozen in liquid nitrogen. X-ray diffraction data were collected from a single crystal at the Life Sciences Collaborative Access Team (LS-CAT) at the Advanced Photon Source (APS).

Diffraction data were processed using HKL2000 (39) (Table 1). Molecular replacement was performed by the program Phaser (40) using the δ(1–66) structure as the initial model. The atomic model was built using PHENIX (41) and COOT (42) and the final structure was refined to 1.8-Å resolution using PHENIX to a R_work_ value of 18.6% and R_free_ of 22.6%. The coordinates were then deposited in the RCSB Protein Data Bank (PDB ID: 5W82). Structure figures were prepared using PyMOL (Schrödinger, LLC).

**TABLE 1:** X-ray data statistics of Orsay δ(1–101)

Parameter	Value^*a*^
Data collection
Space group	P2_1_2_1_2_1_
Unit cell dimensions, Å	a = 55.74, b = 58.22, c = 163.24
Resolution, Å	50–1.8 (1.83–1.8)
Total no. of frames	250
Total no. of reflections	196,759
Unique reflections	49,567
I/σ	12.3 (1.3)
Redundancy	4.0 (3.1)
Completeness, %	98.5 (95.7)
*R*_merge_, %	10.7 (83.3)
CC_1/2_	0.981 (0.685)
Molecular Replacement	
TFZ	49.0
LLG	2237.133
Refinement	
*R*_work_, %	18.6
*R*_free_, %	22.6
Ramachandran plot	
Most favored	98.99%
Generally allowed	1.01%
Disallowed	0

a Numbers in parentheses are for the highest-resolution shell.

### Worm culture and virion purification.

The C. elegans *rde-1* (ne219) V strain (WM27) was maintained at 20°C on NGM plates seeded with OP50 bacteria, per standard protocols (43). Plates were infected with Orsay viral filtrate as previously described (1). Infected worms were then washed to S medium for liquid culture (43), supplemented with additional OP50 and 4% Orsay viral filtrate (vol/vol), and cultured in a 20°C incubating shaker for 5 days.

Infected worm culture was harvested and clarified via centrifugation at 34,500 × *g* for 30 min (Beckman Coulter JA-25.50 rotor). The clarified supernatant was then treated with 1% Triton X-100 (Fisher Scientific) for 1 h at room temperature in an 170-rpm shaking incubator. Virions were subsequently pelleted via ultracentrifugation at 142,000 × *g* for 2 h (Beckman Coulter Type 45 Ti rotor) and resuspended in a buffer consisting of 20 mM Tris–HCl pH 7.8, 0.1% 2-mercaptoethanol, 100 mM NaCl, and 1 mM EDTA. The resuspended virion-containing pellet was then treated with an additional 0.1% Triton X-100 and left overnight at 4°C with gentle rotation, followed by an additional round of ultracentrifugation at 284,000 × *g* for 2 h (Beckman Coulter Type 80 Ti rotor). The resulting virion pellet was then resuspended in the aforementioned buffer and once again treated with an additional 1% Triton X-100 for 1 h at room temperature in an 170-rpm shaking incubator. The resuspension was then layered atop a 25% to 47.5% iodixanol step gradient, prepared with the aforementioned buffer, and subjected to ultracentrifugation at 247,000 × *g* for 16 h (Beckman Coulter SW 41 Ti rotor). The gradient was then fractionated to collect the lower virus-containing band. The resulting sample was subsequently dialyzed through a 30-kDa ultrafiltration column (Millipore Sigma) to remove excess iodixanol and flowed through equilibrated Ni-NTA resin (Thermo Fisher) before being stored at 4°C for subsequent Western blot analysis.

### Electron microscopy.

For negative staining EM, FCF400-Cu grids (Electron Microscopy Sciences) were pretreated by glow-discharge at 5 mA for 1 min as previously described (8). Protein solution (5 μl) was then added onto the grid and allowed to sit for 1 min to allow absorption. Any residual protein solution was then removed from the grids by filter paper blotting. The grids were then rinsed twice with distilled water and stained with freshly prepared 0.75% uranyl formate solution for 1 min. After air drying overnight, grids were examined using a JEOL 1230 high contrast transmission electron microscope at 80 kV. Images were recorded on a Gatan CCD detector.

### Western blotting.

Purified virions were resolved on a 12% SDS-PAGE gel and transferred to polyvinylidene difluoride (PVDF) membrane. After blocking with 5% nonfat milk in Tris-buffered saline (TBS, pH 7.4) containing 0.1% Tween 20 (TBST), membranes were probed with anti-CP antiserum for 1 h at 25°C. Membranes were then washed three times with TBST and incubated with horseradish peroxidase (HRP)-conjugated secondary antibodies for 1 h at 25°C. After washing three times with TBST, immune-reactive bands were detected using SIGMAFAST BCIP/NBT alkaline phosphatase substrate (Sigma). Intensities of CP-δ and CP were measured using Fiji (44).

### Mass spectrometry.

Purified protein samples were diluted to 200 ng/μl with buffer A (2% ACN, 0.1% FA). Liquid chromatography was performed with a Thermo Scientific DIONEX UlitMate 3000 RSLCnano system with a ProFlow Pump block. Buffer A was 2% ACN, 0.1% FA; buffer B was 98% ACN, 0.1% FA. A 15-cm long, 100-μm ID column, packed with 3.5-μm beads with 300-Å pores was used (ZORBAX C3-300SB). The gradient started at 5% B and went to 95% B in 25 min following a 1-μl sample injection. A 1700 V Nanospray Flex source was used with an ion transfer tube temperature of 320°C. Data acquisition was performed by a Thermo Scientific Orbitrap Fusion Lumos. The mass spectrometer (MS) was run in positive mode, with the orbitrap for all scans operating at a resolution of 15,000, a window of 1,000 to 4,000 *m/z*, 5.0e5 AGC, 60% RF, 200 ms max injection time, and 10 microscans.

### δ protein:virus competition assay.

The protein-competition assay was performed using the C. elegans strain SS104 *glp-4(bn2)* I as previously described (8). Briefly, a 2-fold serial dilution of viral filtrate was prepared to determine virus concentration and screened for infectivity. The lowest viral concentration that could maintain a ≥70% infection rate across plates was used for protein competition assays. Approximately 100 synchronized *glp-4(bn2)* L1 stage naive worms were then added to each well of a 96-well plate containing 100 μl of S medium with 1 mM IPTG, 50 ng/μl carbenicillin, *rde-1* RNAi bacteria, Orsay virus, and 0.18 nM test protein (Orsay capsid, Orsay δ, Santeuil δ, or Le Blanc δ). Animals were cultured in a 20°C incubator shaker until reaching the day-3 adult stage. Animals were then transferred from each well to an unseeded NGM plate to count for worms with the transparent intestine symptom, indicating viral infection. A plate with over 50% transparent worms was scored as an infected plate. The percentage of infected plates was calculated to measure viral infectivity.

Wild isolate C. briggsae strain JU1264 was used to test the infectivity of Le Blanc virus and Santeuil virus against δ protein competition. Approximately sixty synchronized wild-type C. briggsae L1 stage worms were added to each well of a 96-well plate containing 100 μl of S medium with OP50 bacteria, Le Blanc virus or Santeuil virus, and 0.54 nM test protein. Animals were cultured in a 20°C incubator shaker for 72 h, washed 4 times with S medium to remove L1 progeny, and supplemented with fresh S medium and OP50 bacteria to a total volume of 100 μl. Worms were then incubated in a 20°C shaker until reaching the day-3 adult stage and transferred to an unseeded NGM plate for counting using the transparent-intestine phenotype as previously mentioned.

### Data availability.

The δ(1–101) structure is available in the Protein Data Bank (PDB) under accession code 5W82.
